# Non-Steroidal Anti-inflammatory Drugs As Host-Directed Therapy for Tuberculosis: A Systematic Review

**DOI:** 10.3389/fimmu.2017.00772

**Published:** 2017-06-30

**Authors:** Vera M. Kroesen, Matthias I. Gröschel, Neil Martinson, Alimuddin Zumla, Markus Maeurer, Tjip S. van der Werf, Cristina Vilaplana

**Affiliations:** ^1^Carl-von-Ossietzky University Oldenburg, Oldenburg, Germany; ^2^Experimental Tuberculosis Unit (UTE), Fundació Institut Germans Trias i Pujol (IGTP), Universitat Autònoma de Barcelona (UAB), Badalona, Catalonia, Spain; ^3^Department of Pulmonary Diseases & Tuberculosis and Internal Medicine, University Medical Center Groningen, University of Groningen, Groningen, Netherland; ^4^Department of Infectious Diseases, University Medical Center Groningen, University of Groningen, Groningen, Netherland; ^5^Perinatal HIV Research Unit, University of Witwatersrand, Johannesburg, South Africa; ^6^Division of Infection and Immunity, University College London (UCL), London, United Kingdom; ^7^National Institute of Health Research’s Biomedical Research Centre, University College London Hospitals NHS Foundation Trust, London, United Kingdom; ^8^Department of Microbiology, University College London Hospitals NHS Foundation Trust, London, United Kingdom; ^9^Division of Therapeutic Immunology, Department of Laboratory Medicine, Karolinska Institutet, Stockholm, Sweden; ^10^Centro de Investigación Biomédica en Red de Enfermedades Respiratorias (CIBERES), Madrid, Spain

**Keywords:** non-steroidal anti-inflammatory drugs, host-directed therapies, tuberculosis, systematic review, infectious diseases

## Abstract

Lengthy, antimicrobial therapy targeting the pathogen is the mainstay of conventional tuberculosis treatment, complicated by emerging drug resistances. Host-directed therapies, including non-steroidal anti-inflammatory drugs (NSAIDs), in contrast, target host factors to mitigate disease severity. In the present Systematic Review, we investigate whether NSAIDs display any effects as therapy of TB and discuss possible mechanisms of action of NSAIDs as adjunctive therapy of TB. Ten studies, seven preclinical studies in mice and three clinical trials, were included and systematically reviewed. Our results point toward a beneficial effect of NSAIDs as adjunct to current TB therapy regimens, mediated by decreased lung pathology balancing host-immune reaction. The determination of the best timing for their administration in order to obtain the potential beneficial effects needs further investigation. Even if the preclinical evidence requires clinical evaluation, NSAIDs might represent a potential safe, simple, and cheap improvement in therapy of TB.

## Introduction

Tuberculosis (TB) is a chronic infectious disease caused by *Mycobacterium tuberculosis* (Mtb) causing a wide spectrum of disease in humans (1). The current approach to treat TB entails antimicrobial drugs that target mycobacteria (2). Drugs to target host immune function rather than focusing on the bacteria have been proposed as adjuvants to classic antimicrobial treatment, with the advantage of not selecting TB drug resistance (3). The wide range of these host-directed therapies (HDTs)—including NSAIDs (4)—act on host immune effectors to achieve decrease in host-destructive pathology; potentially leading to clinical improvement, decreased morbidity, and mortality. Pathologic immune reactions in the host, such as insufficient or excessive inflammatory response leading to severe tissue damage are considered a major cause of failure of current TB treatment (5).

Neutrophils represent a protective immune response in early infection through secretion of oxidizing and hydrolytic agents targeted at the bacteria (6). While this neutrophil-dominated inflammation is beneficial in the acute infection, it can be detrimental in the context of chronic infection (6). Excessively aggressive immune response in active, chronic TB disease destroys host tissue leading to necrosis and cavitation, facilitating spread of the bacilli (7). Attenuating excessive host inflammatory response in active TB might thus be beneficial during treatment and for disease outcome (6). NSAIDs, based on their anti-inflammatory effect, could act on attenuating excessive (neutrophil-mediated) inflammation in active TB disease (4, 6, 8).

The aim of this review was to investigate whether NSAIDs are a useful HDT candidate for TB. With this purpose, we systematically reviewed the published manuscripts on the preclinical and clinical effects of NSAIDs when used as therapy of TB, alone or in combination with commonly used anti-TB drugs or BCG vaccination.

## Methods

We used the 2009 PRISMA guidelines to systematically review PubMed for studies published until January 2017 that evaluate NSAIDs as therapy of TB. The following search terms were used: “tuberculosis” OR “mycobacterium tuberculosis” combined with drug names of the NSAID group: ((tuberculosis OR (mycobacterium tuberculosis)) AND (ibuprofen OR aspirin OR naproxen OR etoricoxib OR indomethacin OR meloxicam OR celecoxib OR diclofenac (DCL) OR oxyphenbutazone OR carprofen) NOT “review” [publication type]).

Two investigators independently reviewed the title and abstract of all identified publications for relevance. Studies were deemed relevant when they met the following criteria: clinical or animal studies, English language, published on Pubmed from 1950 through January 2017, study subjects are TB patients (sensitive and resistant, pulmonary and extra-pulmonary), who received a drug from the NSAID-group. We excluded *in vitro*, open-label, and self-reporting studies as well as Case Reports. The reference list of included publications was furthermore screened for relevant publications. Disagreements between investigators were resolved by discussion. The full text of the included studies was reviewed for relevant information to answer the question whether NSAIDs as HDTs are beneficial during TB treatment. Information was systematically collected according to predetermined criteria, i.e., drug, study, experimental approach, TB patients/mouse strain, sample size, intervention, and outcome.

Two investigators independently reviewed the full text of all identified publications. For clinical trials (CTs), the Jadad Scoring System was used to evaluate quality (9).

## Results

A total of ten relevant studies were identified and included for systematic review (Table 1). The full texts of all ten studies were retrieved for in depth analysis. The three CTs (10–12) were of high quality, according to Jadad scoring.

**Table 1:** An overview on the data acquisition from relevant studies for systematic review.

Drug	Reference	Experimental approach	Patients/mouse strain	Sample size	Intervention	Outcome
Aspirin and Ibuprofen	Vilaplana et al. (13)	Murine model of *active* TB: mice infected with Mtb were treated with ibuprofen when their health started to deteriorate	C3HeB/FeJ mice	60	Infection of mice i.v. with Mtb H37Rv. Oral ibuprofen treatment 80mg/kg/day *week 3 or week 4 after infection	Reduction in the no. and size of lung lesions, significantly decreased bacillary load in lungs and increased survival in ibuprofen group

	Byrne et al. (14)	Murine model: mice infected with Mtb were treated with Isoniazid (H) in combination with aspirin or ibuprofen	BALB/c mice	30	Aerosol infection of mice with Mtb H37Rv. *1 day after infection, 5 days/week/4 weeks oral aspirin or ibuprofen (respectively, 10, 20, and 40 mg/kg) alone, or isoniazid (25 mg/kg) in combination with aspirin or ibuprofen, respectively	Aspirin or ibuprofen alone: no significant effect on CFU counts. Isoniazide (H) alone: reduction of CFU counts. Ibuprofen with (I): further decrease of CFU counts. Aspirin with (I): increased CFU counts (compared to H only)

	Byrne et al. (15)	Murine model: mice infected with Mtb were treated with aspirin, ibuprofen or pyrazinamid (Z) alone or in combination in initial phase of disease	BALB/c mice	30	Aerosol infection of mice with Mtb H37Rv. *1 day after infection, 5 days/week/4 weeks oral aspirin (20 mg/day) or ibuprofen (20 mg/day) or pyrazinamid (150 mg/day), alone, or pyrazinamid in combination with aspirin or ibuprofen	Aspirin or ibuprofen alone: no significant effect on CFU counts. Pyrazinamide alone: reduction of CFU counts. Aspirin or ibuprofen with Z: further reduction of CFU counts

	Petty et al. (12)	Clinical trial (CT): assessment of serum uric acid concentrations in TB patients treated with Z (and ethionamide and H) before, during and after additional treatment with low-dose aspirin; three patients presented with arthralgias	TB patients; no laboratory signs of renal impairment, not treated with urate retaining drugs, no special diet	11	Enrolled patients had received Z for 3 days to 18 months; oral acetylsalicylic acid (AAS) 2.4 g/day was added for three consecutive days. Serum uric acid was measured: on 2 days during Z-only treatment before AAS was added; on day 2 and 3 of additional AAS-treatment; 2 days after end of additional AAS-treatment	Low-dose aspirin (2.4 g/day 3 days) significantly lowered serum uric acid to almost normal levels (mean pre-AAS: 8.67 mg/100 ml; AAS 4.56 mg/100 ml; post-AAS 8.43 mg/100 ml) in all 11 patients; when taken out after 3 days, levels immediately returned to pre-AAS treatment levels; in one patient additional AAS treatment was continued for 8 weeks; after which serum uric acid concentration remained low; in three patients AAS attenuated mild arthralgias

	Horsfall et al. (11)	CT: patients with arthralgia during treatment with (Z) were treated with allopurinol or aspirin	Patients with arthralgia during treatment with (Z)	60	Anti-arthralgia treatment for 8 weeks: *n* = 18 aspirin 2.4 mg/day, *n* = 23 allopurinol 200 mg/day, *n* = 19 placebo only (randomly allocated)	Most patients improving joint symptoms and signs. More in aspirin and placebo groups, most rapid in aspirin group; only in aspirin group, mean serum uric acid concentration lower during treatment than before

	Schoeman et al. (10)	CT: children with probable diagnosis of TBM (*at least clinical signs and CFU changes typical for TBM) were treated with low-or high-dose aspirin or a placebo added to anti-TB treatment	Children with diagnosis of probable* TBM	159 enrolled, *146* included, rest excluded because pre-treated with aspirin, but included in open-label trial	*n* = 50: placebo group (daily sorbitol); *n* = 47: low-dose aspirin group (daily 75 mg); *n* = 49: high-dose aspirin group (daily 100 mg). Monitoring for side effects	8 deaths: 1 placebo group, 2 low-dose aspirin group, 3 high-dose aspirin group, 2 open-label group (1 death in may have been due to aspirin). Motor outcome: no significant differences, but of 9 children who developed hemiplegia, none in the high dose aspirin group. Cognitive outcome: Griffiths test (after 6 months of treatment) no significant differences

Indomethacin	Shroff et al. (16)	Murine model: mice i.p. immunized with *M. vaccae* and pre-treated with indomethacin were infected with Mtb	BALB/c and Swiss white mice	50	6 i.p. (50 μg/mouse) i.p. injections at 12 h intervals of indomethacin, 12 h after last dose i.p. immunization with *M. vaccae*. Control 1: no pre-treatment, but immunization. Control 2: no immunization	Immunized, indomethacin pre-treated mice did show immunization response (CPE 0.18). Immunized, non-pre-treated mice didn’t show immunization response (CPE 0.02)

	Hernandez et al. (17)	Murine model: mice with induced lung granulomas of TB were treated with indomethacin or Cyclophosphamid (INN)	BALB/c mice	?	Culture filtrate proteins (CFP) obtained from Mtb. Immunization of mice with CFP or mBSG. After 10 days intra-tracheal infection with CFP- or mBSG-coated beads (control). For testing effect of indomethacin and INN: 5 mg/kg/day i.p. administration of indomethacin *during the whole experimental procedure*, 20 mg/kg i.p. injection of INN 1 day before infection	CFP-granulomas significantly bigger than mBSA-granulomas, CD8+ T-cells dominating, reduced DTH and antibody titers. Upon treatment with INN or Indomethacin; significant reduction of granuloma size, increase in DTH and antibody titers. mBSA-granulomas, composed quite equally of CD8+ and CD4+ T-cells, did not alter antibody titers

Diclofenac (DCL)	Dutta et al. (18); *in vivo* part discussed here, *in vitro* part not further mentioned here	Murine model: mice were injected with 10 mg/kg DCL and then challenged with a 50 median lethal dose of potent clinical isolate Mtb H37Rv102	Swiss white mice	Groups of 20, how many groups?	Parenterally infection with 0.05 ml suspension (0.5 mg homogenized culture KLM, Kirchers Liquid medium) equaling c < 9 × 10^9^ CFU). Of each group, 10 mice were DCL-treated (10 mg/kg/day) for 6 weeks, 10 not treated as control	DCL-treated mice: less tubercles and none in the lungs (compared to control). Histophathological section of liver: significantly less infiltrations in DCL-treated mice. Smears for acid-fast bacilli (by Z-N strain) positive for all 10 untreated mice, only in 4 DCL-treated mice. Mtb recovery in subculture from 5 control mice and 1 DCL-treated mice (*P* < 0.01)

	Dutta et al. (19); *in vivo* part discussed here, *in vitro* part not further mentioned here	Murine model: mice infected with Mtb were treated for with either DCL or Streptomycin (S) alone, or in combination.	Swiss albino mice	210	I.v. infection of mice with Mtb H37Rv; 5 treatment groups: (1) day 1 control (baseline values for spleen weight), (2) untreated control, (3) DCL-treated (10 μg/g/day, orally), (4) STM-treated (150 μg/g/day, subcutaneously), (5) DCL+ STM-treated (STM 1 h after DCL); for 4 weeks	Treatment with either DCL or STM alone significantly reduced bacterial counts in lungs and spleen and mean spleen weight, and increased survival (compared to control). Simultaneous administration further decreased bacterial counts and spleen weight and increased survival significantly (also compared to STM only)

Total number	10					

### Ibuprofen and Aspirin

Ibuprofen acts as an unselective cyclooxygenase (COX)-inhibitor. COX control the production of lipid mediators derived from arachidonic acid, such as prostaglandins (PGs), resolvins, lipoxins, leukotriene, and thromboxanes (TXAs). Three studies were found for Ibuprofen (13–15).

Acetylsalicylic acid (AAS) is a non-selective COX-inhibitor, which irreversibly inhibits COX-enzymes. In addition to its anti-inflammatory effects, it is widely used in prevention of cardiovascular disease and strokes (20) due to its cardio-protective effects mediated by irreversible inhibition of TXA A2 production in nuclei-free platelets. Five studies were identified for Systematic Review of aspirin (10–12, 14, 15).

Vilaplana et al. treated Mtb infected C3HeB/FeJ mice—in a murine model of active TB—with 80 mg/kg/day ibuprofen (13). Ibuprofen increased survival and significantly decreased the number and size of lung lesions consistent with decreased bacillary loads in the lungs. Histologic examination revealed excessive neutrophilic infiltration in the control group, which was found to be alleviated in the Ibuprofen-group (13).

Byrne et al. treated Mtb-infected mice with either the first-line anti-TB drugs pyrazinamid (Z) or isoniazid (H), or with the NSAIDs ibuprofen or aspirin, alone or in combination with H. Treatment was started 1 day after infection (14). Compared to controls, Z alone reduced bacterial counts significantly in lungs and spleens. Ibuprofen with H enhanced this effect, while with aspirin reduced it. Ibuprofen and aspirin alone did not have any significant effect on bacterial counts compared to controls but enhanced the clearance of Mtb in tissues achieved by H. The authors suggest the use of ibuprofen rather than aspirin as adjunctive analgesic in TB-treatment (14). However, another *in vivo* study by Byrne et al. showed that both ibuprofen and aspirin enhance the effect of Z and might be evaluated to shorten therapy (15). Mtb-infected mice were treated with aspirin (20 mg/day), ibuprofen (20 mg/day) or Z (150 mg/day) alone or Z in combination with either aspirin or ibuprofen, from one day after infection. Z alone reduced bacterial counts compared to controls, and this effect was enhanced by both aspirin and ibuprofen (15).

Two CT were found supportive of the synergistic effect of aspirin and Z: short-term, low-dose aspirin was shown to prevent the side effect of hyperuricaemia in Z-treatment of TB (12). Petty et al. treated TB patients who received anti-TB treatment with Z (and H and ethionamide) with additional oral low-dose aspirin 2.4 g/day for three consecutive days. Short-term, low-dose aspirin significantly lowered serum uric acid concentrations to almost normal levels in all enrolled patients, and in three patients who presented with mild arthralgias, AAS controlled them. In line with the results from Petty et al., aspirin has been vsuggested to ameliorate arthralgia in Z-treatment of TB (11). Horsfall et al. followed patients with arthralgia during common TB-therapy containing Z, administered with additional anti-arthralgia treatment: aspirin 2.4 mg/day or allopurinol 200 mg/day or a placebo (11). Most patients in all three treatment groups reported improvement, though the improvement was greater and faster in in the aspirin group (11).

Schoeman et al. conducted a double-blind, randomized phase II CT to evaluate the effect of aspirin-treatment as an add-on to standard anti-TB treatment (and prednisone) on mortality and morbidity of children with a probable diagnosis of tuberculous meningitis (TBM) (10). The primary endpoints were survival, motor and cognitive outcome and the number of serious adverse events. Patients were randomly grouped into three adjunctive treatment groups (Table 2). There was no effect of aspirin on the outcome of hemiplegia when it was already present at admission, though of nine children who developed hemiplegia, none was in the high dose aspirin group. In total, eight children died, relatively equally distributed to all three treatment groups, one death possibly caused by aspirin treatment (75 mg/day) (10). Results did not show any significant beneficial or adverse effect of aspirin. The high-dose aspirin group in this study turned out to have worse baseline characteristics at start, with more hemiparesis at baseline, which might have distorted the results (10).

**Table 2:** Treatment groups in Schoeman et al. (10).

Group	Dosage (no. of patients)	Anti-tb treatment	Monitoring for side-effects
Placebo	Daily sorbitol (*n* = 50)	Daily H, R, E (20 mg/kg/day)	Daily: GIT- and bleeding disorders
Low-dose acetylsalicylic acid (AAS)	75 mg/day (*n* = 47)	Daily Z (40 mg/kg/day)	Weekly: liver function, Reye-syndrome
High-dose AAS	100 mg/day (*n* = 49)	Daily prednisone (4 mg/kg)	

### Indomethacin

Indomethacin is a non-selective COX-inhibitor and a non-salicylate NSAID similar to ibuprofen. Two studies were identified which furthermore provide special insights into apparent immune-modulatory properties of NSAIDs-like indomethacin (16, 17).

A study by Hernandez-Pando et al. evaluated immunosuppressive phenomena based on T-cell imbalance in the TB-granuloma and its treatability with cyclophosphamide and indomethacin (17). Cyclophosphamide is an alkylating agent used during cancer-treatment and for autoimmune-disorders. Mice were immunized against Mtb and 10 days after immunization, infected with Mtb-coated beads to induce granuloma formation. To evaluate their effect, 5 mg/kg/day indomethacin was administered intraperitoneally (i.p.) during the whole experimental procedure and 20 mg/kg cyclophosphamide was injected i.p. 1 day before infection. Control granulomas induced by mBSA-coated beads were composed quite equally of CD8+ and CD4+ T-cells and did not alter antibody titers against Mtb. TB granulomas were significantly bigger, mainly composed of CD8+ cytotoxic T-cells and showed a drop in DTH-reaction (delayed type hypersensitivity reaction) and antibody titers against Mtb. This imbalance was reversible upon administration of indomethacin or cyclophosphamide with significant reduction of granuloma size, increase in DTH and antibody titers. Conclusively, an imbalance in T-cells within TB-induced granulomas mainly being composed of CD8+ cytotoxic T-cells, and few CD4+ T-helper cells down-regulates cell-mediated and humoral immunity to Mtb locally. This is reversible upon treatment with indomethacin (17).

Shroff et al. found that indomethacin enhance the response of mice to intraperitoneal immunization with *M. vaccae* (16). Mice were pre-treated with indomethacin and then immunized with *M. vaccae*. After 48 h DTH-reaction was tested, the non-pre-treated did not show any reaction of immunization, the pre-treated mice did show immunization responses. Thus, the non-responder state of mice to *M. vaccae* by i.p. route of immunization was corrected by pre-treatment with indomethacin (16).

### Diclofenac

Diclofenac is an unselective COX-inhibitor with anti-bacterial properties. Anti-mycobacterial action has been shown in two animal studies (18, 19).

Dutta et al. treated Mtb-infected mice with DCL shortly after infection (18), the DCL-treated mice showing less lesions and lower bacillary loads (measured by positive smears for acid-fast bacilli and positive subculture for Mtb) compared to controls (18).

A second study by Dutta et al. showed synergistic activity of DCL in combination with Streptomycin (S) (19). Mice were infected with Mtb and treated with DCL, S or S + DCL in combination (19). Both, treatment with S or DCL alone decreased bacterial counts in lungs and spleens and mean spleen weight, and increased survival. All these effects were increased by the simultaneous administration of S + DCL (19).

## Discussion

**Figure 1:**
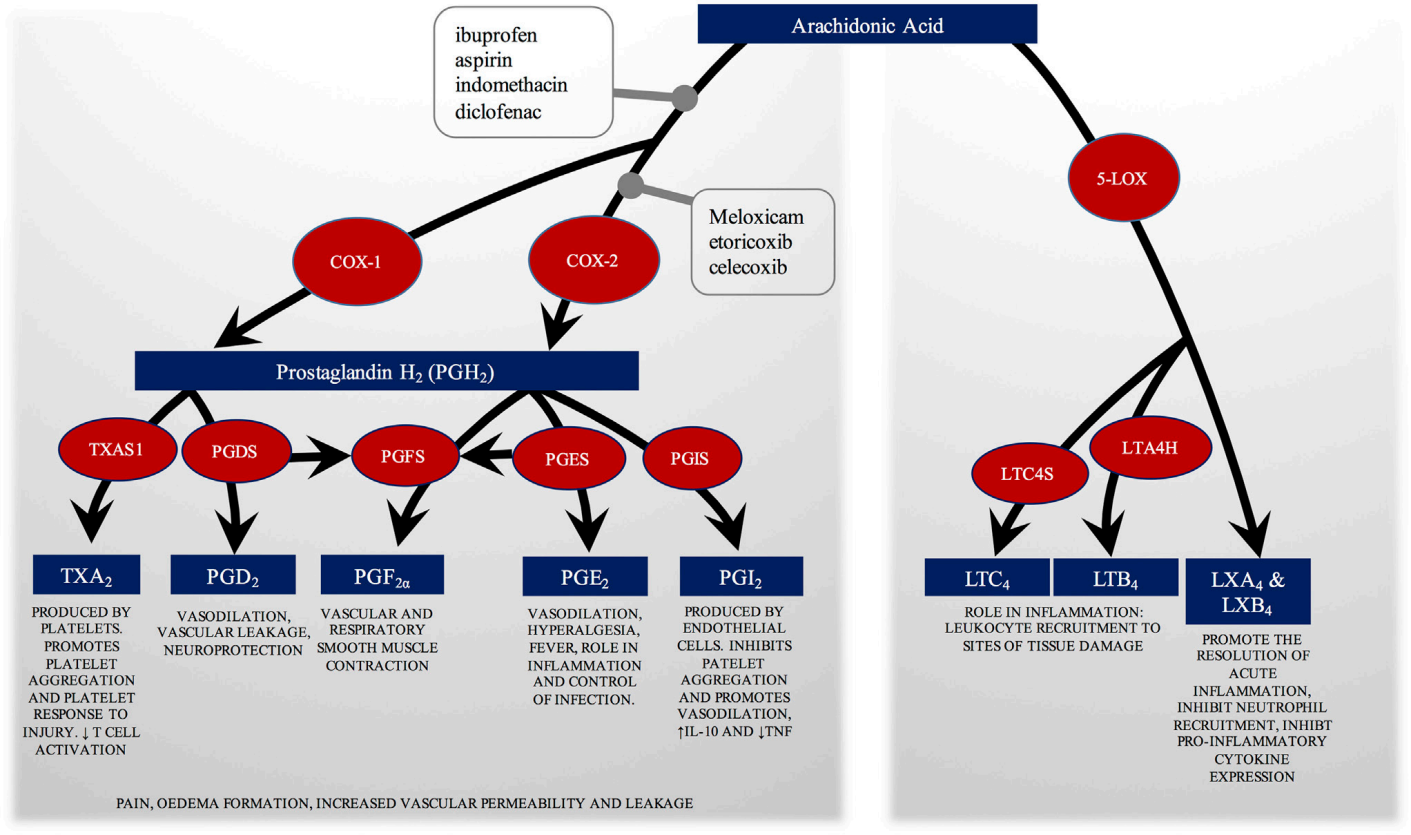
COX and 5-LOX pathways of eicosanoid biosynthesis from arachidonic acid. The physiological role of each eicosanoid and eicosanoid group is explained. The figure shows where the non-steroidal anti-inflammatory drugs (NSAIDs) evaluated in this review have an effect, blocking both COX enzymes or the COX-2 selectively. By inhibiting the COX enzymes, NSAIDs inhibit prostaglandin synthesis. They also promote a switch to arachidonic acid oxidation by 5-LOX. COX, cyclooxygenase; LOX, lipoxygenase; TXA, thromboxane; TXAS, thromboxane synthase; PG, prostaglandin; PGDS, PGD synthase; PGFS, PGF synthase; PGES, PGE synthase; PGIS, PGI synthase; LT, leukotriene; LTC4S, LTC_4_ synthase; LTA4H, LTA_4_ hydrolase; LX, Lipoxin.

Our results are based on the search for specific NSAID drug names, we include only English written articles and exclude open-label and self-reporting studies as well as Case Reports. These restrictions in the search strategy impose a selection bias on results. Our review combines results, expressed as various endpoints, from ten different studies, where experimental approach, patients, sample size, and intervention differ (see Table 1). Figure 1 shows two of the main pathways (COX and 5-LOX) of eicosanoid biosynthesis from arachidonic acid, and where the NSAIDs have an effect.

Though in total 10 studies were identified for Systematic Review, the anti-inflammatory effect of NSAIDs was only investigated as an endpoint (granuloma size in lungs) by two studies, which furthermore assessed differing characteristics of assessed granulomas (13, 17). However, all seven reported animal studies provide evidence for a clear beneficial effect of NSAIDs in TB (expressed by various endpoints), and two of three reported CT found mild beneficial effects of aspirin alleviating side-effects of Z (11, 12). Included animal studies did not report side-effects of treatment with NSAIDs. The three CTs reported no severe side effects except two, including one death, which underline the importance of monitoring for risk factors and side-effects when investigating NSAIDs in general (10). To conclude, our results from seven animal studies point to a clear beneficial effect of NSAIDs in TB, but the individually reported beneficial effects need more investigation and, when supported, consequently clinical confirmation.

Regarding the animal studies included, need to remember that humans respond with a wide spectrum to infection with Mtb, from innately resistant to extremely susceptible (21). Key factor for survival of Mtb in the human host is the induction of lung necrosis, which enables spreading of the bacilli (21). The immunologic response to Mtb infection in mice is largely genetically determined (21). None of the currently available models in mice represents the whole spectrum of human TB, but specific models match specific phenotypes and pathways of inflammation of human TB (21). The two most commonly employed inbred laboratory mouse strains [C75BL/6 (B6) and BALB/c mice] do not develop necrotic lesions (21), in spite of developing chronic asymptomatic infection. There are animal models available which effectively mimic lung necrosis of the susceptible human individual, i.e., the C3HeB/FeJ model mimics both necrosis and cavitation (21). Therefore, the choice of the specific animal model matching the specific research question is of great importance. Vilaplana et al. employ C3HeB/FeJ mice to evaluate the effect of ibuprofen on lung pathology in TB (13). Four of the seven animal studies assessed employed BALB/c mice (Table 1) to assess ibuprofen and aspirin as adjunct to commonly used TB drugs (14, 15), to evaluate the effect of indomethacin on vaccination with *M. vaccae* (16) and to study the effect of indomethacin on granuloma composition (specifically T-cell balance) (17). Dutta et al. employ Swiss white mice to assess the anti-bacterial effect of DCL (18, 19).

According to the results of our Systematic Review, NSAIDs can be beneficial if given as co-therapy during TB therapy. This protective effect is possibly mediated by their anti-inflammatory properties, by enhancing activity of TB-antibiotics and possibly by own directly bactericidal activity.

Excess recruitment of granulocytes (primarily neutrophils) leads to uncontrolled inflammation and exacerbation of TB disease (6, 22). Infection of macrophages leads to a neutrophilic-dominated inflammatory response (8, 13), which—in absence of sufficient Mtb-elimination—leads to an excessive, neutrophilic-dominated, inflammatory response, and disease exacerbation: eventually, the host succumbs to infection, before the adaptive immune response can fully start working (7, 22, 23). NSAIDs could impact on these interactions by reducing the destructive inflammatory response *via* inhibition of PGE2 in granulocytes as well as by enhancing effectors of adaptive immunity such as IFN-γ (4, 24, 25). In agreement with this hypothesis, Vilaplana et al. found that neutrophils are of major responsibility for chronic inflammation and tissue damage associated with granulomas (8), and that the anti-inflammatory properties of NSAIDs can mitigate this phenotype (8, 13). Findings by Hernandez-Pando and Shroff et al. point to NSAIDs possibly balancing cell-mediated, adaptive immune response to Mtb. Hernandez-Pando et al. found that indomethacin can balance CD8+- and CD4+-ratio in TB-granulomas reducing excessive inflammation and morbidity (17).

The reduction of prostaglandin E2 (PGE2) is considered one mechanistic explanation underlying the anti-inflammatory benefit of NSAIDs in the context of Mtb infection (4), as PGE2 inhibits phagocytosis and bacterial killing at late stage of Mtb-infection. However, a protective effect of PGE2 on the mitochondrial membrane against Mtb in macrophages has been described, suggesting it to be beneficial at early stages (4). Another explanation is NSAIDs action against PG-production in polymorphonuclear leukocytes in late stage Mtb-infection, as all these cells are highly implicated in the formation and maintenance of granulomas (4, 7). They all possess COX-2 activity producing PGs and therefore drive the chronic inflammation and tissue damage associated with granulomas in late Mtb-infection (4). For all these, an accurate evaluation of the timing of NSAIDs as an add-on in TB-treatment is warranted. The beneficial effect of NSAIDs is based on their ability to reduce excessive and tissue-damaging inflammation mainly in late stage infection. They may control liquefaction of the granulomas and consequently enhance healing (4, 13); thus might have positive effects in patients with active TB, usually present after months of infection.

On the other hand, patients with severe TB-disease are at higher risk of developing thrombosis and stroke, due to an hypercoagulable state in advanced stages of the diseases (26). Low-dose aspirin is proven to have protective effects in other patients at risk of developing ischemic stroke (20). Though in the reviewed CT by Schoeman et al. anti-coagulative therapy with low-dose aspirin did not show any beneficial effects in childhood TBM, different baseline characteristics of the patients enrolled might have distorted results (10).

There is evidence that suggests an immune-mediated pathoetiology of vascular events in TB patients. Vasculitis in TBM is morphologically similar to other immune-mediated vasculitis (27) and the anti-inflammatory effect of glucocorticoids has successfully been used in treatment of TBM (28) as well as TB-IRIS (TB-associated Immune Reconstitution Inflammatory Syndrome) (29). Likewise, a recent open-label CT has evaluated aspirin in combination with corticosteroids in TBM and found a reduction of strokes and mortality (30). Indeed, hypercoagulability cannot be uncoupled from inflammatory processes. Increased cytokine levels are associated with progression of atherosclerosis and development of ischemia in patients with stable angina (31), injured epithelia secrete more cytokines and cause local inflammation which fuels atherosclerosis (31). Atherosclerosis and hypercoagulability are cytokine-mediated and thus an inflammatory process, this could explain the benefit of corticosteroids in TBM and TB-IRIS. The effect of NSAIDs in states of inflammatory hypercoagulability of TB should further be investigated.

With rising drug-resistances against commonly employed anti-TB drugs, new add-on drugs need to be employed, enhancing anti-mycobacterial efficacy to avoid the need to increase doses to potentially toxic levels (31). *In vivo* data by Dutta et al. suggests an enhancing effect of DCL on the TB-antibiotic S as well as an own anti-mycobacterial effect of DCL (18). *In vitro* data equally demonstrated some inhibitory effect of many NSAIDs on mycobacteria by targeting various biological functions in the bacilli (32) (see Table 3). Aspirin reduces the expression of genes for energy metabolism in bacteria (33).

**Table 3:** Non-steroidal anti-inflammatory drugs (NSAIDs) acting as anti-tubercular non-antibiotics (including *in vitro* studies).

Drug	Study	Implications
Ibuprofen	Elvers, antibacterial activity of the anti-inflammatory compound ibuprofen. 1995. *in vitro* (34)	Inhibition of Protein translation initiation (inhibition of bacterial initiation factor 2) (35, 36)
Ibuprofen and Carprofen	Guzman, antitubercular specific activity of ibuprofen and the other 2-arylpropanoic acids using the HT-SPOTi whole-cell phenotypic assay. 2013. *in vitro* (36)	Inhibition of DNA replication and repair (acting on ‘bacterial sliding clamp’ = DNA-polymerase III β) (37)
Aspirin	Schaller, salicylate reduces susceptibility of *Mycobacterium tuberculosis* to multiple anti-tuberculosis (TB) drugs. 2002. *in vitro* (33)	Down-regulating bacterial transcription and translation especially genes for energy production (38)
Diclofenac (DCL)	Dutta, *in vitro* and *in vivo* antimycobacterial activity of antiinflammatory drug, DCL sodium. 2004. *in vivo and in vitro* (18)	Against Gram-positive and Gram-negative bacteria possibly by inhibition of bacterial DNA synthesis (39, 40)
	Dutta, Activity of DCL used alone and in combination with streptomycin against *Mycobacterium tuberculosis* in mice. 2007. *in vivo and in vitro* (19)
	Sriram, synthesis, *in vitro* and *in vivo* antimycobacterial activities of DCL acid hydrazones and amides. 2006. *in vitro* (41)
Celecoxib (Cox-II Selective)	Kalle, inhibition of bacterial multidrug resistance by celecoxib, a cyclooxygenase-2 inhibitor. 2011. *in vitro* (42)	Inhibition of bacterial drug-resistance by inhibiting efflux mechanism (regulation of bacterial MDR-1 efflux pumps) (43)
	Salunke, design and synthesis of novel anti-TB agents from the celecoxib pharmacophore. 2015. *in vitro* (44)	
Oxyphenbutazole	Gold, non-steroidal anti-inflammatory drug sensitizes *Mycobacterium tuberculosis* to endogenous and exogenous antimicrobials. 2012. *in vitro* (45)	Multifactorial: inhibition of bacterial enzymatic reactions by affecting flavins and thiols (32)
NSAIDs bound to metal-complexes	Chiniforoshan, anti-inflammatory drugs interacting with Zn (II) metal ion based on thiocyanate and azide ligands: synthesis, spectroscopic studies, DFT calculations and antibacterial assays. 2014. *in vitro* (46)	
	Kovala-Demertzi, Organotin meclofenamic complexes: synthesis, crystal structures, and antiproliferative activity of the first complexes of meclofenamic acid—novel anti-TB agents. 2009. *in vitro* (47)	
Total	11	

Non-steroidal anti-inflammatory drugs as add-on could leverage the efficacy of commonly used antibiotic anti-TB drugs, but if displaying intrinsic bactericidal activity, development of drug-resistance against NSAIDs themselves would have to be considered (19). However, most anti-tubercular non-antibiotics so far have displayed *in vitro* MICs from 10–25 µg/ml, which are concentrations beyond clinical achievability (48). However, intracellular concentration of NSAIDs by macrophages may enhance killing: by phagocytosis, they might concentrate drugs in question more than 10× in the phagocytosed target Mtb (48). The bactericidal dose might be achievable *in situ*. Therefore, possible benefits of various NSAIDs as anti-tubercular non-antibiotics implied by *in vitro* data (see Table 3) require *in vivo* and consequently clinical confirmation.

Non-steroidal anti-inflammatory drugs as potential HDT would be considered as adjunctive in TB-treatment and would be intended to be administered in addition to common anti-TB treatment regimens. Thus, interaction mechanisms with commonly used anti-TB drugs and alterations of pharmacodynamics need to be evaluated. So far, no published study has evaluated interaction of NSAIDs with the current full four-drug regimen used for human drug-susceptible TB.

Salicylate was found to have *in vitro* adverse effects in combination with the first line TB-drugs H, R, E, and Streptomycin (S) (33). In contrast, in *in vivo* combination with the first line TB-drug Z it showed synergistic effect (15). Z is converted to pyrazinoic acid and needs an acidic pH for full efficacy, which increases as the metabolism of the mycobacterium decreases. This is why it acts best against dormant, persistent Mtb (49). Microarray data suggest that salicylates cause a de-energized status in Mtb by down-regulating translation and transcription of genes for energy production (38). This is supported by the fact that aspirin enhanced the effect of Z *in vivo*, but not *in vitro* (15). This does however not explain the synergistic effect of ibuprofen in combination with Z. Byrne et al. suggest that an anti-inflammatory mechanism *via* reduction of PGs was less likely, as this would antagonize the acidic pH built in inflammation needed for action of Z (15). However, since the efficacy of Z is not pH-dependent despite a slightly acidic pH optimum, the excessive inflammation might reduce its efficacy, which is the case for the other anti-TB drugs as well.

Currently, four CTs are ongoing to evaluate the effect of NSAIDs during TB treatment. The anti-inflammatory properties of NSAIDs seem of potential benefit in TB-IRIS (29): an ongoing trial is evaluating the benefit of meloxicam in TB-IRIS (NCT02060006). Based on the findings by Vilaplana et al. (13), a pilot study is currently conducted in Georgia, to assess the potential safety and efficacy of ibuprofen as an add-on in the treatment of pre-XDR and XDR-TB patients (NCT02781909). Moreover, there is a pilot study on the way, which aims to evaluate the benefit of low-dose (75 mg/day) and high-dose aspirin: 1 g/day (much higher than in Schoeman et al. (100 mg/day) (10) as an adjunctive to anti-TB treatment of HIV-negative adults with TBM (NCT02237365). Shroff et al. found that the non-responder state of mice to *M. vaccae* by i.p. route of immunization was corrected by pre-treatment with indomethacin (16); and this strategy should be evaluated for other therapeutic vaccines as well. In this sense, there is an ongoing CT (NCT02503839) evaluating the concurrent administration of a COX-2 inhibitor (etoricoxib) and a therapeutic vaccine in addition to conventional TB-treatment in MDR-TB. *In vitro* and *in vivo* data showed that DCL and many other NSAIDs have direct bactericidal properties (see Table 3) (18). A CT is evaluating the bactericidal *ex vivo* activity of celecoxib (a COX-selective NSAID) against Mtb in whole blood from healthy volunteers (NCT02602509).

In conclusion, and despite open questions that should be addressed in further research and the well-known side effects, NSAIDs potentially have beneficial effect in treatment of TB (8, 13). Even if most of the evidence is limited to preclinical studies and consequently clinical studies are warranted for confirmation of safety and efficacy, NSAIDs could be a secure, simple, and (depending on the NSAID drug) a cheap adjunction for future TB (including MDR/XDR-TB)-treatment regimens.

## Author Contributions

All authors made substantial contributions to the conception or design of the work. VK and MG contributed to the acquisition, analysis, interpretation of data for the work. All authors drafted the work, revised it critically for important intellectual content, and gave final approval of the version to be published; and agreed to be accountable for all aspects of the work in ensuring that questions related to the accuracy or integrity of any part of the work are appropriately investigated and resolved.

## Conflict of Interest Statement

The authors declare that the research was conducted in the absence of any commercial or financial relationships that could be construed as a potential conflict of interest.
